# Illuminating the genetics of complex human diseases

**DOI:** 10.1186/1753-6561-6-S6-O4

**Published:** 2012-10-01

**Authors:** Michael C Schatz

**Affiliations:** 1 Simons Center for Quantitative Biology. Cold Spring Harbor Laboratory, Cold Spring Harbor, NY, USA

## 

In 2007, Zhao *et al. *[1] proposed a unified genetic theory of sporadic and inherited autism spectrum disorders to explain the complex familial patterns observed. The model explains why most families have a low risk of autistic children with an overall incidence rate of approximately 1 in 150 births, but a small minority of families have a 50% risk for male offspring. Their model links these two risk classes by their genetic origins: sporadic autism in low-risk families is mainly caused by highly penetrant spontaneous mutations in autism-related genes, whereas inherited autism in high risk families is mainly caused by unaffected parents carrying a causative mutation that is transmitted in a dominant fashion to their offspring. The evidence for the model was based on the available genotyping assays that showed an elevated rate *of de novo* copy number variants (CNVs) in children with autism spectrum disorders.

Since then, we and other collaborators have been following up with a high-resolution exome sequencing study of 2,800 families from the Simons Simplex Collection to pinpoint the genetic components of the disorder. Unlike the early studies that could only detect large copy number events spanning tens or hundreds of thousands of basepairs across multiple genes, our new study has power to examine single nucleotide and indel mutations within individual genes. From our preliminary analysis of approximately 350 of these families [2], as well as the reports of three other groups, we have collected strong evidence for the role of'likely gene-disrupting'(LGD) mutations (nonsense, splice site and frame shifts), with affected individuals having twice as many LGDs compared to unaffected siblings, and five 'double hit' genes (*CHD8*, *DYRK1A*, *KATNAL2*, *POGZ*, *SCN2A*) having two de novo mutations in unrelated individuals. From this analysis, we estimate approximately 400 genes are targets of autism spectrum disorders. Interestingly, from the gene set we have already identified, we have discovered a strong association between the targets LGD mutations in autism and in vivo targets of the RNA-binding translational regulator FMRP (encoded by *FMR1*), which results in Fragile X Syndrome when silenced or mutated. The large number of samples in the study necessitated that we develop a high performance parallel sequence analysis pipeline that could scale to the large volume of data and make use of local disk storage. We were able to make use of several existing tools for the preliminary analysis (BWA, SAMTools, GATK, etc), and we also developed additional components for genotyping within a family and across the population using a multinomial statistical approach. This includes a novel sequence analysis algorithm for discovering insertion & deletion variants using a localized sequence assembly approach that is superior to standard mapping algorithms. We continue to refine the algorithms to improve scalability, sensitivity, and specificity, and are beginning to apply it towards analyzing the genomes of families with other complex cognitive disorders.
